# The Relative Contribution of Jawbone and Cheekbone Prominence, Eyebrow Thickness, Eye Size, and Face Length to Evaluations of Facial Masculinity and Attractiveness: A Conjoint Data-Driven Approach

**DOI:** 10.3389/fpsyg.2018.02428

**Published:** 2018-12-05

**Authors:** Justin K. Mogilski, Lisa L. M. Welling

**Affiliations:** ^1^Department of Psychology, University of South Carolina Salkehatchie, Walterboro, SC, United States; ^2^Department of Psychology, Oakland University, Rochester, MI, United States

**Keywords:** face preference, face shape, masculinity, attractiveness, conjoint analysis, data-driven, digital manipulation

## Abstract

Recent work demonstrates the methodological rigor of a type of data-driven analysis (i.e., conjoint analysis; CA), which accounts for the relative contribution of different facial morphological cues to interpersonal perceptions of romantic partner quality. This study extends this literature by using a conjoint face ranking task to predict the relative contribution of five sexually dimorphic facial shape features (jawbone and cheekbone prominence, eyebrow thickness, eye size, face length) to participants’ (*N* = 922) perceptions of facial attractiveness and sex-typicality (i.e., masculinity/femininity). For overall partner attractiveness, eyebrow thickness and jawbone prominence were relatively more salient than cheekbone prominence and eye size. Interestingly, masculinized (i.e., thicker) eyebrows were marginally more attractive for female than male faces, particularly within a long-term mating context. Masculinized jawbone prominence was more attractive for male than female faces, and feminized jawbone prominence was more attractive for female than male faces. For perceptions of masculinity, eyebrow thickness, jawbone prominence, and facial height were relatively more salient than cheekbone prominence and eye size, although facial height was more important for female than male faces, and jawbone prominence was marginally more important for male than female faces. These findings highlight the prominence of eyebrows, the jawline, and facial height during perception of facial attractiveness and masculinity – though it should be noted that many of these differences were small to moderate in effect size. Findings are interpreted in the context of prior research, and future directions for studying why these facial traits exhibit superior signaling capacity are discussed.

## Introduction

Facial morphological cues (e.g., shape, color, and texture) are indicators of underlying physiology (Stephen et al., 2009; Little et al., 2011b; Jones et al., 2012). From these cues, humans can accurately predict certain physical and psychological qualities (e.g., an individual’s health, physical attractiveness, trustworthiness) that are significant to partner selection and social judgment (e.g., Zebrowitz, 2011; Todorov et al., 2015). These qualities can be assessed by observers from facial photographs at first acquaintance, and significantly impact employment decisions, mate selection, friendship, and other key aspects of social interaction (e.g., Petrican et al., 2014; Walker and Vetter, 2016; Funk et al., 2017). Face perception researchers have studied how these cues are processed during interpersonal evaluation by digitally manipulating photographic facial cues and presenting these images to third-party raters. These manipulations predictably alter perceptions of attractiveness, dominance, sex-typicality (i.e., masculinity/femininity), health, trustworthiness, and other social attributes (for a review, see Todorov et al., 2008; Little et al., 2011a). However, this research has tended to focus on individual facial cues in isolation, and debate is now turning to the relative contributions of these cues to social perception (Scott et al., 2010; Stephen et al., 2012; Mogilski and Welling, 2017).

Data-driven models are becoming more valued within scientific face perception research for their capacity to account for a broader array of structural and configural facial features, and the contributions of those specific features to social perception, compared to traditional methods alone (Todorov et al., 2011; Zhang et al., 2018). Early studies that used digital face stimuli to alter and assess person perception (e.g., Perrett et al., 1999; Penton-Voak et al., 2001) measure the influence of perceptually distinct facial cues (e.g., symmetry and sexual dimorphism) by digitally altering one feature while experimentally or statistically controlling for variation in other features. Although this work makes compelling contributions to the literature by reducing confounds, it tells us little about how collections of traits are evaluated in combination. Recent data-driven techniques have overcome this limitation by permitting distinct clusters of features to be altered simultaneously (e.g., Mogilski and Welling, 2017; Stephen et al., 2017; Jones, 2018). For example, Stephen et al. (2017) recorded participants’ physiological health (i.e., blood pressure, BMI, percent body fat) and regressed these measures onto variation in facial morphology. These measures were then subjected to factor analysis to identify which clusters of facial features predict variation in health. Participants were asked to change the appearance of potential romantic partners’ facial photographs to fit their preference using sliders that incrementally altered faces along each health dimension, thereby digitally manipulating the constellation of facial features naturally associated with variation in these health indices. Similarly, Jones (2018) recorded participants’ self-reported health and implemented a Brunswick lens model to assess which facial cues are utilized to assess health, and which cues are valid indicators of health. Photographs were subsequently manipulated according to whichever cues were most utilized and valid. Compared to prior techniques, these methods identify facial cues and manipulate them via digital transformations that are based on a broad collection of naturalistic features, rather than from artificially restricted parameters based on theory alone.

Although these models have been particularly useful for initially exploring, identifying, and simulating the facial cues that contribute to person perception and social decision-making, they are limited in their capacity to assess the relative contribution of several concurrently altered facial cues to holistic perception of those faces. In the studies noted above (Stephen et al., 2017; Jones, 2018), digital transformations were applied to facial images and rated sequentially rather than concurrently. For example, Stephen et al. (2017) asked participants to manipulate a series of faces to appear as healthy as possible by manipulating apparent BMI, blood pressure, and body fat, but each of these dimensions was manipulated independently and rated across separate trials. Jones (2018) manipulated two features concurrently (i.e., averageness and color), but asked participants to assess stimuli of different combinations (e.g., high averageness, low color) across separate line-ups of faces. These methods allow researchers to examine preference for feature combinations, but they are limited by how many feature combinations may be examined at the same time without separating them into separate trials or experimental conditions.

### Conjoint Analysis

Conjoint analysis (CA) provides a convenient way to overcome this design challenge. CA is a multivariate, data-driven analysis used in marketing research (e.g., Gustafsson et al., 2007) that has recently been adapted to study human mate preferences (Mogilski et al., 2014; Mogilski and Welling, 2017). Generally, CA is used to assess how individuals make trade-offs among multiple attributes when evaluating “whole” units that comprise those attributes. For example, CA is often used to evaluate which attributes of a product are most important during consumer purchasing decisions by having consumers rank several versions of the product, where each version is composed of a unique combination of product attributes. Mogilski and Welling (2017) first used this technique to examine the relative salience of three facial cues (i.e., sexual dimorphism, color, and symmetry) during romantic partner perception. Compared to other methods, this technique allows researchers to present sets of faces wherein each face is altered by several different features at once. Participants rank these sets on some metric (e.g., their attractiveness as a romantic partner) and CA provides measures of the relative contribution of each feature to participants’ overall ranking decisions. Using this technique, Mogilski and Welling (2017) found that facial shape masculinity/femininity was relatively more important than both symmetry and color cues to health during participants’ evaluations of potential romantic partners’ facial photographs. However, presenting individuals with multiple versions of mates who vary across several different traits is but one potential use of CA. This technique can also be used to explore preferences for feature variations that relate to a single construct. Specifically, CA can investigate how several traits that contribute toward a single construct impact rater’s perceptions of that construct.

### Current Study

The present study contributes to current face perception literature by using CA to assess the relative contributions of several facial shape cues to perceptions of romantic partner attractiveness and masculinity. Previous research (e.g., Scheib et al., 1999; Penton-Voak et al., 2001; Koehler et al., 2004; Thornhill and Gangestad, 2006; Apicella et al., 2008) has identified several prominent shape cues that contribute to perceptions of sexual dimorphism (i.e., features that differ statistically between male and female faces): eyebrow prominence, cheekbone prominence, eye size, facial height, and jawbone prominence. Among these features, eyebrow prominence, facial height, and jawbone prominence are all reliably larger in men (i.e., are positively related to a masculine face shape and negatively related to a feminine face shape), whereas cheekbone prominence and eye size were reliably larger in women (i.e., are positively related to a feminine face shape and negatively related to a masculine face shape). Identification of the specific features that impact the overall measured sexual dimorphism of a particular individual is important for perceptual research, but no research to date has investigated how these individual features are prioritized relative to one another with respect to mate choice or with respect to the overall evaluation of a person’s masculinity/femininity.

This study assessed whether individuals prioritize certain sexually dimorphic facial cues of partner quality (i.e., eyebrow prominence, cheekbone prominence, eye size, facial height, and jawbone prominence) when evaluating the attractiveness of same- and opposite-sex individuals’ facial photographs as long- and short-term romantic partners. Furthermore, it investigated whether individuals prioritize any of these specific features when ranking different versions of the same individual by perceived masculinity. Given that face perception is, in part, due to part-based information processing (Schwaninger et al., 2004; McKone and Yovel, 2009), it is possible that the part-worth value of some facial features is weighted more heavily compared to others during partner perception. Similarly, some features may be more salient than others within different mating contexts (e.g., long- versus short-term; Buss and Schmitt, 1993; Gangestad and Simpson, 2000). Examining how these features are prioritized within long- (i.e., committed) versus short-term (i.e., purely sexual) mating contexts may explain the specific signal value of the distinct facial shape cues that contribute to person perception, and thereby reveal which features are most important to perceptions of attractive facial cues (e.g., Thornhill and Gangestad, 1999; Gangestad and Scheyd, 2005; Puts, 2010). Indeed, developing techniques that improve the accuracy of models that estimate psychological and physiological qualities from facial information (see Hu et al., 2017; Todorov, 2017) is a critical future direction in face perception research (see Jack and Schyns, 2017, for a review). Moreover, because this is the first study to manipulate individual features rather than whole faces by masculinity and femininity, the contribution of each manipulation to perceptions of facial masculinity will reveal interesting information about how we process this trait, as well as serve to further verify the computer graphics manipulations. Finally, this study will expand prior findings that used CA to study face perception (Mogilski and Welling, 2017). Because this study found that sexually dimorphic shape cues were more important than color and symmetry cues (Cohen’s d ∼0.60), this study sought to examine which shape features were driving this effect. That is, this study examines whether some shape features signal relatively more information about partner quality (i.e., attractiveness; masculinity/femininity) than others.

## Materials and Methods

### Participants

Participants (*N* = 922, 250 male; age: *M* = 20.22 years, *SD* = 3.53; range = 18–51) were recruited from a university in the mid-western United States and various social media outlets (e.g., Facebook, Reddit, Twitter). The majority of participants were White (78.4%; Black 9.5%, Asian 5.5%, Hispanic/Latino 2.3%, “Other” 4.3%), roughly half reported currently being single (49% versus 51% reported being in a romantic relationship), and the majority reported being exclusively heterosexual (91.3%; 6.5% bisexual, 2.2% exclusively homosexual).

### Stimuli

Using well-established methods (e.g., Jones et al., 2005; Little et al., 2007; Welling et al., 2008), composite male and female faces were generated by averaging the shape, color, and texture of a group of 60 Caucasian adult male faces and a group of 60 Caucasian adult female faces. Each composite served as the base image for a set of 19 photographs that varied exclusively by a series of objective, composite-based image transformations (detailed below). Up to five distinct facial characteristics were transformed per photograph variation: eyebrow prominence, cheekbone prominence, eye size, facial height, and jawbone prominence. These features are sexually dimorphic and vary with perceptions of facial attractiveness (Keating, 1985; Scheib et al., 1999; Penton-Voak et al., 2001; Baudouin and Tiberghien, 2004). To permit CA of participants’ photograph rankings, each of the 19 photograph variations were planned using an orthogonal array generated with IBM SPSS 21, which is constructed according to a standard formula drawn from statistical reference material. A fractional-factorial design was used to minimize the number of photograph variations that participants were required to rank (Hair et al., 1995). This design generates the fewest number of profiles needed to estimate the contribution of each of the five facial characteristics to overall face evaluation. Each of the five facial features were assigned three possible levels (i.e., feature masculinization, unaltered, or feature feminization), indicating which transformations would be applied to each photograph. This produced an orthogonal array of 16 photograph variations, whereby each variation possessed a unique combination of the five facial characteristics. For example, a face might have masculinized eyebrow prominence, feminized cheekbone prominence, unaltered eye size, feminized facial height, and masculine jawbone prominence. Three additional holdout images (for a total of 19 photographs) were included to test the validity of the CA utility estimates (see Hair et al., 1995; utility estimates and holdout profiles are defined in more detail in the Results section below). All participants ranked the same 19 images constructed based on the orthogonal array.

**FIGURE 1:**
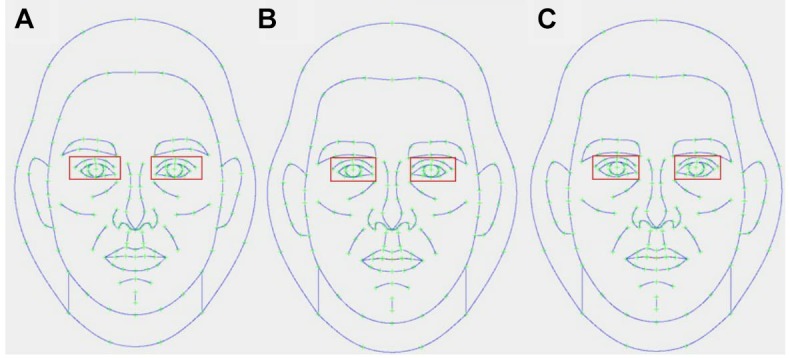
Example of how composite templates for individual features were created. The above image shows specifically how eye size was manipulated, but the same technique was also independently used for the other manipulated features. **(A)** Is a female composite image and **(B)** is a male composite image. To create a male composite with feminine eye size, all points within the red square in **(B)** were altered to match corresponding points in **(A)**. **(C)** Is a male composite with feminine eye size [i.e., all points within the red squares match **(A)** (female composite), whereas all points outside the red squares match **(B**) (male composite)].

**FIGURE 2:**
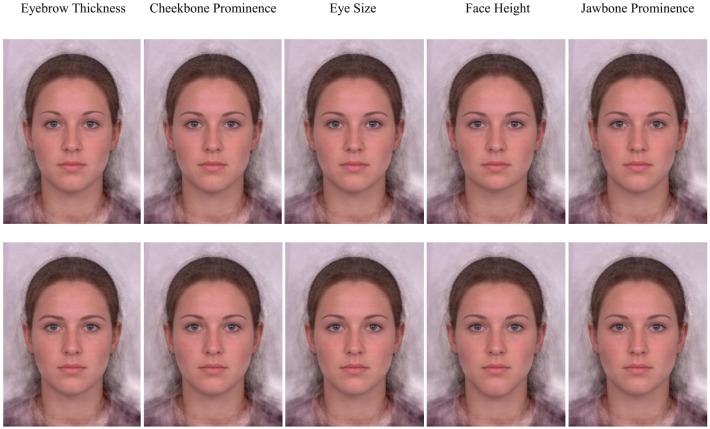
Examples of each independent feature manipulation applied to the female base composite image. Feature manipulations are organized into columns. Feminized features are presented in the top row and masculinized features are presented in the bottom row.

**FIGURE 3:**
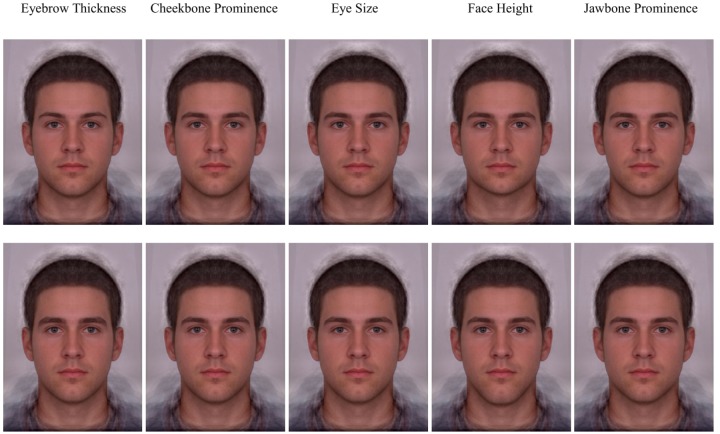
Examples of each independent feature manipulation applied to the male base composite image. Feature manipulations are organized into columns. Feminized features are presented in the top row and masculinized features are presented in the bottom row.

**FIGURE 4:**
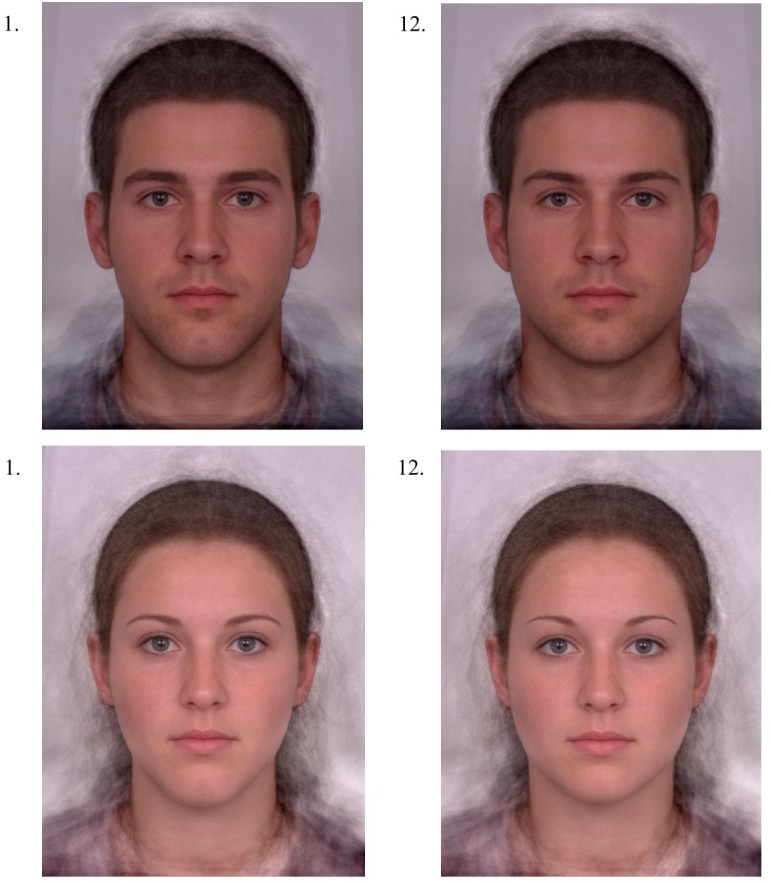
Examples of composite male (top) and female (bottom) images to which one or more of the five digital transformations were applied according to an orthogonal array (see Table 1). Numerical labels correspond to the “Imagine Variation” number listed in Table 1. Base composite images were borrowed from previous work (e.g., Perrett et al., 1998; Penton-Voak et al., 1999; Jones et al., 2005; Welling et al., 2007).

To alter specific facial features, image transformation methods used in prior work (e.g., DeBruine et al., 2006; Welling et al., 2007, Welling et al., 2008) were adapted to target individual features rather than whole faces. Specifically, rather than apply composite-based transformations holistically to base images (i.e., to the whole face), 5 male and 5 female composite images were first created, whereby each composite image possessed one feature (i.e., eyebrow prominence, cheekbone prominence, eye size, facial height, or jawbone prominence) of the opposite-sex, but that was otherwise sex-typical. Thus, for each composite image, the points that correspond to individual features (e.g., eye size) were altered such that they matched the position of those same points on the opposite-sex composite (see Figure 1 for an example). These composite images were then applied to same-sex base images by taking 50% of the linear differences in 2D shape between the applicable altered composites (e.g., Figure 1, image C) and the original same-sex composites (i.e., Figure 1, image A for women, image B for men) and adding to or subtracting from corresponding points on the base image. Figures 2, 3 demonstrate the complete array of masculine and feminine manipulations individually applied to base female (Figure 2) and male (Figure 3) composite faces. These transformations were then applied to base images (i.e., the original, unaltered composite images) according to the orthogonal array (see Table 1). In other words, facial features were manipulated as per previous research (e.g., Welling et al., 2007, Welling et al., 2008) except that individual features were independently manipulated and then concurrently applied to the same face (see examples in Figure 4). Although no study has manipulated individual facial features in this way, holistic facial manipulations using these techniques have been shown to influence perceptions of masculinity and femininity in the predicted directions (DeBruine et al., 2006; Welling et al., 2007).

**Table 1:** Orthogonal array of facial transformations and the respective image variations to which they were applied.

Image variation	Eyebrow	Cheekbone	Eye Size	Face Height	Jawbone
1	Unaltered	Masculine	Feminine	Masculine	Feminine
2	Unaltered	Feminine	Masculine	Masculine	Masculine
3	Masculine	Feminine	Unaltered	Masculine	Unaltered
4	Masculine	Unaltered	Masculine	Feminine	Feminine
5	Masculine	Masculine	Feminine	Unaltered	Masculine
6	Feminine	Masculine	Masculine	Unaltered	Unaltered
7	Feminine	Unaltered	Masculine	Masculine	Masculine
8	Masculine	Unaltered	Feminine	Masculine	Unaltered
9	Unaltered	Masculine	Masculine	Feminine	Unaltered
10	Feminine	Feminine	Feminine	Feminine	Masculine
11	Masculine	Masculine	Unaltered	Feminine	Masculine
12	Feminine	Masculine	Unaltered	Unaltered	Masculine
13	Unaltered	Unaltered	Unaltered	Unaltered	Masculine
14	Masculine	Feminine	Masculine	Unaltered	Feminine
15	Masculine	Masculine	Masculine	Masculine	Masculine
16	Masculine	Masculine	Masculine	Masculine	Masculine
17 (Holdout)	Masculine	Feminine	Masculine	Masculine	Unaltered
18 (Holdout)	Masculine	Unaltered	Unaltered	Masculine	Feminine
19 (Holdout)	Masculine	Masculine	Masculine	Masculine	Feminine

*The number listed in the “Image variation” column corresponds to each image’s numerical label in Figure 4.*

### Procedure

All experimental materials were presented using Qualtrics. After indicating their consent, participants provided demographic information (i.e., age, sex, ethnicity, relationship status, and sexual orientation) and then completed a series of four face ranking tasks. For each task, participants were presented with each of two sets (one male, one female) of nineteen digital facial photographs. Participants were asked to rank the images within each set relative to one another twice: once according to their preference for a long-term relationship and once according to their preference for a short-term relationship. Long- and short-term relationships were defined for participants as follows:

Long-term relationship: *You are looking for the type of person who would be attractive in a long-term relationship. Examples of this type of relationship would include someone you may want to move in with, someone you may consider leaving a current partner to be with, and someone you may, at some point, wish to marry (or enter into a relationship on similar grounds as marriage).*

Short-term relationship: *You are looking for the type of person who would be attractive in a short-term relationship. This implies that the relationship may not last a long time. Examples of this type of relationship would include a single date accepted on the spur of the moment, an affair within a long-term relationship, and the possibility of a one-night stand.*

Participants were instructed to rank same-sex photographs according to how they believed a heterosexual person of the opposite-sex would rank them. The order in which the face ranking tasks and photographs within sets were presented was randomized.

## Results

CA was performed to assess the relative importance of each of the five facial features in participants’ ranking decisions. CA produces importance values, which indicate a feature’s overall contribution to how profiles are ranked (e.g., the overall importance of cheekbone prominence, eye size, etc.), and part-worth utility estimates, which indicate the relative importance of each level within each trait (i.e., masculinization, feminization, unaltered). In other words, importance values reveal which features are weighted most heavily relative to others during ranking decisions, but not the direction of preference within any given feature (e.g., whether eyebrow prominence is more important for ratings of attractiveness than cheekbone prominence, but not whether masculine, unaltered, or feminine eyebrow prominence is preferred). On the other hand, utility estimates reveal the importance of the manipulation within a trait (e.g., preference for masculine, unaltered, versus feminine eyebrow prominence). Importance values and part-worth utility estimates were calculated for each set of faces.

Participants’ rankings of holdout profiles were accurately predicted by the utility estimates (all *τ* = 1.00) for both attractiveness and masculinity assessments. Holdout profiles are image variations with unique facial characteristic combinations that are ranked alongside the original 16 profile, but which are not used to generate importance values or utility estimates. The attractiveness or masculinity of a holdout profile can be calculated using the model generated from participants’ rankings of the other 16 images. That is, the utility estimates for each characteristic (e.g., masculinized face height, feminized eye size, etc.) can be summed within each image variation to give an overall estimated attractiveness or masculinity score for that image. How well the score for each holdout profile predicts participants’ rankings of those profiles relative to each of the other 16 profiles is represented by the tau coefficient. In other words, tau represents of how well the utility estimates predict participants’ rankings of the holdout profiles relative to the other 16 profiles.

For each of the following analyses, participant gender was included as an additional variable. All interactions with gender were nonsignificant even before Bonferroni correction (adjusted critical *p* = 0.01; all *p*s > 0.08). Therefore, participant gender was excluded from our report below.

### Attractiveness Ratings

#### Importance Values

looseness2 A 2(sex of face [male, female]) × 2(relationship context [long-term, short-term]) × 5(facial attribute [eyebrow thickness, cheekbone prominence, eye size, face height, jawbone prominence]) repeated-measures ANOVA was used to examine differences in importance values for each facial attribute in male and female faces ranked for desirability as long- and short-term mates. All *post hoc* analyses and pairwise comparisons were adjusted using Bonferroni correction (critical *p* = 0.01). There was a main effect for facial attribute, *F*(4, 3684) = 23.41, *p* < 0.001, η^2^ = 0.03. Importance values for eyebrow thickness (*M* = 21.28, *SD* = 6.62) were not significantly different from face height (*M* = 20.31, *SD* = 7.61, *p* = 0.114) or jawbone prominence (*M* = 20.48, *SD* = 5.66, *p* = 0.155), but both eyebrow thickness and jawbone prominence were greater than cheekbone prominence (*M* = 18.43, *SD* = 4.42, *p* < 0.001, *d* = 0.31, *d* = 0.29) and eye size (*M* = 19.49, *SD* = 5.36, *p* < 0.001, *d* = 0.19, *d* = 0.12) when ranking faces for attractiveness. Likewise, importance values were greater for face height than for cheekbone prominence (*p* < 0.001, *d* = 0.19).

There was also a significant interaction between sex of face and facial attribute, *F*(4, 3684) = 6.97, *p* < 0.001, η^2^ = 0.01. Importance values for eye size were greater for male faces (*M* = 20.09, *SD* = 7.28) than for female faces (*M* = 18.90, *SD* = 7.12), *t*(921) = 3.76, *p* < 0.001, *d* = 0.12, whereas importance values for face height were greater for female faces (*M* = 20.90, *SD* = 9.98) than for male faces (*M* = 19.73, *SD* = 8.36), *t*(921) = 3.45, *p* = 0.001, *d* = 0.12. There was also a significant interaction between relationship context and facial attribute, *F*(4, 3684) = 2.39, *p* = 0.049, η^2^ = 0.003. Importance values for eyebrow thickness were greater for a long-term (*M* = 21.71, *SD* = 8.26) compared to short-term (*M* = 20.95, *SD* = 8.17) relationship context, however, this was not significant after Bonferroni correction, *t*(921) = 2.04, *p* = 0.042, *d* = 0.08.

#### Utility Estimates

Five 2(sex of face [male, female]) × 2(relationship context [long-term, short-term]) × 3(attribute level [masculinized, unaltered, feminized]) repeated-measures ANOVAs were used to examine differences in utility estimates for each level of each facial attribute in male and female faces ranked for desirability as long- and short-term mates. All *post hoc* analyses and pairwise comparisons were adjusted using Bonferroni correction (critical *p* = 0.017).

##### Eyebrow thickness

There was a main effect for attribute level, *F*(2, 1842) = 27.37, *p* < 0.001, η^2^ = 0.03. Utility estimates were greater for masculinized (*M* = 0.25, *SD* = 0.95) than for unaltered (*M* = -0.12, *SD* = 1.03, *p* < 0.001, *d* = 0.23) and feminized (*M* = -0.13, *SD* = 1.13, *p* < 0.001, *d* = 0.21) eyebrow thickness. This was moderated by a significant interaction with sex of face, *F*(2, 1842) = 3.81, *p* = 0.022, η^2^ = 0.004. Utility estimates for masculinized eyebrow thickness were greater for women (*M* = 0.33, *SD* = 1.35) than for men (*M* = 0.17, *SD* = 1.19), *t*(921) = 2.85, *p* = 0.004, *d* = 0.10. This was further moderated by a three-way interaction, *F*(2, 1842) = 3.14, *p* = 0.044, η^2^ = 0.003. For female faces, utility estimates for masculinized eyebrows were higher in a long- (*M* = 0.42, *SD* = 1.75) versus short-term (*M* = 0.25, *SD* = 1.68) context, *t*(921) = 2.39, *p* = 0.017, *d* = 0.11, though this was only marginally significant after Bonferroni correction. There were no other significant main effects or interactions (all *F* < 1.96, all *p* > 0.141).

##### Cheekbone prominence

There was a main effect for attribute level, *F*(2, 1842) = 30.63, *p* < 0.001, η^2^ = 0.03. Utility estimates were greater for masculinized (*M* = 0.21, *SD* = 0.79) than unaltered (*M* = -0.11, *SD* = 0.78, *p* < 0.001, *d* = 0.25) and feminized (*M* = -0.11, *SD* = 0.90, *p* < 0.001, *d* = 0.21) cheekbones. There were no other significant main effects or interactions (all *F* < 2.09, all *p* > 0.124).

##### Eye size

There was a main effect for attribute level, *F*(2, 1842) = 58.21, *p* < 0.001, η^2^ = 0.06. Utility estimates were higher for unaltered (*M* = 0.23, *SD* = 0.78) than masculinized (*M* = 0.09, *SD* = 0.91, *p* = 0.006, *d* = 0.11) and feminized (*M* = -0.32, *SD* = 1.07, *p* < 0.001, *d* = 0.30) eye size. Estimates were also higher for masculinized compared to feminized eye size (*p* < 0.001, *d* = 0.25). There was also a significant interaction between relationship context and attribute level, *F*(2, 1842) = 3.37, *p* = 0.035, η^2^ = 0.004. Utility estimates for masculinized eye size were greater in a short- (*M* = 0.15, *SD* = 1.04) versus long-term (*M* = 0.04, *SD* = 1.05) context, *t*(921) = -2.37, *p* = 0.018, *d* = 0.08, whereas unaltered eye size was preferred in a long- (*M* = 0.29, *SD* = 1.21) compared to short-term (*M* = 0.16, *SD* = 1.24) context, *t*(921) = 2.33, *p* = 0.020, *d* = 0.01, however, both were nonsignificant after Bonferroni correction. There were no other significant main effects or interactions (all *F* < 2.18, all *p* > 0.113).

##### Face height

There was a main effect for attribute level, *F*(2, 1842) = 26.88, *p* < 0.001, η^2^ = 0.03. Utility estimates were greater for unaltered (*M* = 0.24, *SD* = 1.07) versus masculinized (*M* = -0.10, *SD* = 0.84, *p* < 0.001, *d* = 0.21) and feminized (*M* = -0.14, *SD* = 1.06, *p* < 0.001, *d* = 0.24) face height. This was moderated by a significant interaction with sex of face, *F*(2, 1842) = 68.42, *p* < 0.001, η^2^ = 0.07. Utility estimates for masculinized face height were greater for male (*M* = 0.25, *SD* = 1.65) than female (*M* = -0.45, *SD* = 1.65) faces, *t*(921) = 9.71, *p* < 0.001, *d* = 0.30. Likewise, estimates for feminized face height were lower for male (*M* = -0.43, *SD* = 1.45) than female (*M* = 0.15, *SD* = 1.50) faces, *t*(921) = -8.60, *p* < 0.001, *d* = 0.29. There were no other significant main effects or interactions (all *F* < 1.24, *p* > 0.290).

##### Jawbone prominence

There was a main effect for attribute level, *F*(2, 1842) = 101.85, *p* < 0.001, η^2^ = 0.10. Utility estimates were greater for masculinized (*M* = 0.26, *SD* = 0.88, *p* < 0.001, *d* = 0.40) and feminized (*M* = 0.17, *SD* = 0.84, *p* < 0.001, *d* = 0.36) compared to unaltered (*M* = -0.43, *SD* = 1.02) jawbone prominence. This was moderated by a significant interaction between sex of face and attribute level, *F*(2, 1842) = 10.34, *p* < 0.001, *η^2^* = 0.01. Utility estimates for masculinized jawbone prominence were greater for male (*M* = 0.36, *SD* = 1.15) than for female (*M=* 0.16, *SD* = 1.12) faces, *t*(921) = 4.19, *p* < 0.001, *d* = 0.14, whereas estimates for feminized jawbone prominence were greater for female (*M* = 0.28, *SD* = 1.19) than for male (*M* = 0.06, *SD* = 1.21) faces, *t*(921) = -3.88, *p* < 0.001, *d* = 0.13. There were no other significant main effects or interactions (all *F* < 1.30, all *p* > 0.273).

### Masculinity Ratings

#### Importance Values

A 2(sex of face [male, female]) × 5(facial attribute [eyebrow thickness, cheekbone prominence, eye size, face height, jawbone prominence]) repeated-measures ANOVA was used to examine differences in importance values for each facial attribute in male and female faces ranked for masculinity. There was a main effect for facial attribute, *F*(4, 3684) = 17.61, *p* < 0.001, η^2^ = 0.02. Importance values for eyebrow thickness (*M* = 21.09, *SD* = 8.65), face height (*M* = 21.13, *SD* = 10.23), and jawbone prominence (*M* = 20.38, *SD* = 7.49) were not significantly different, but each was significantly greater than cheekbone prominence (*M* = 18.34, *SD* = 6.47; *d* = 0.23; *d* = 0.21; *d* = 0.21, respectively) and eye size (*M* = 19.07, *SD* = 6.98; *d* = 0.16; *d* = 0.15; *d* = 0.11, respectively) (all *p*s < 0.001). This was moderated by a significant interaction with sex of face, *F*(4, 3684) = 5.11, *p* < 0.001, η^2^ = 0.01. Importance values for face height were greater for female (*M* = 22.09, *SD* = 13.18) than for male (*M* = 20.17, *SD* = 11.76) faces, *t*(921) = 4.06, *p* < 0.001, *d* = 0.14. By contrast, importance values for jawbone prominence were greater for male (*M* = 20.90, *SD* = 9.74) than for female (*M* = 19.86, *SD* = 10.21) faces, though this difference was only marginally significant after Bonferroni correction, *t*(921) = 2.39, *p* = 0.017, *d* = 0.08.

#### Utility Estimates

Five 2(sex of face [male, female]) × 3(attribute level [masculinized, unaltered, feminized]) repeated-measures ANOVAs were used to examine differences in utility estimates for each level of each facial attribute in male and female faces ranked for perceived masculinity.

##### Eyebrow thickness

There was a main effect for attribute level, *F*(2, 1842) = 77.97, *p* < 0.001, η^2^ = 0.08. Utility estimates were greater for masculinized (*M* = 0.43, *SD* = 1.29) than unaltered (*M* = 0.08, *SD* = 1.26, *p* < 0.001, *d* = 0.17) and feminized (*M* = -0.51, *SD* = 1.46, *p* < 0.001, *d* = 0.39) eyebrow thickness. There were no other significant main effects or interactions (all *F* < 0.88, all *p* > 0.417).

##### Cheekbone prominence

There was a main effect for attribute level, *F*(2, 1842) = 10.73, *p* < 0.001, η^2^ = 0.01. Utility estimates were greater for masculinized (*M* = 0.17, *SD* = 1.09) than for feminized (*M* = -0.07, *SD* = 1.19, *p* = 0.001, *d* = 0.09) and unaltered (*M* = -0.11, *SD* = 1.10, *p* < 0.001, *d* = 0.11) cheekbone prominence. There was also a significant interaction between sex of face and attribute level, *F*(2, 1842) = 3.17, *p* = 0.042, η^2^ = 0.003. Utility estimates for feminized cheekbone prominence were greater for female (*M* = 0.02, *SD* = 1.64) than for male (*M* = -0.15, *SD* = 1.64) faces, though this was only marginally significant after Bonferroni correction, *t*(921) = 2.34, *p* = 0.019, *d* = 0.08.

##### Eye size

There was a main effect for attribute level, *F*(2, 1842) = 29.53, *p* < 0.001, η^2^ = 0.03. Utility estimates were greater for masculinized (*M* = 0.26, *SD* = 1.02) than for unaltered (*M* = 0.00, *SD* = 1.23, *p* < 0.001, *d* = 0.15) and feminized (*M* = -0.26, *SD* = 1.32, *p* < 0.001, *d* = 0.25) eye size. Estimates were also greater for unaltered than for feminized eye size (*p* = 0.002, *d* = 0.11). There were was no other significant main effects or interactions (all *F* < 0.91, *p* > 0.401).

##### Face height

There was a main effect for attribute level, *F*(2, 1842) = 147.61, *p* < 0.001. η^2^ = 0.14. Utility estimates were greater for masculinized (*M* = 0.72, *SD* = 1.57) than for unaltered (*M* = -0.05, *SD* = 1.12, *p* < 0.001, *d* = 0.34) and feminized (*M* = -0.66, *SD* = 1.49, *p* < 0.001, *d* = 0.49) face height. Similarly, estimates were greater for unaltered compared to feminized face height (*p* < 0.001, *d* = 0.28). There was also a significant interaction between sex of face and attribute level, *F*(2, 1842) = 3.10, *p* = 0.045, η^2^ = 0.003. Utility estimates for masculinized face height were greater for female (*M* = 0.81, *SD* = 2.14) than for male (*M* = 0.63, *SD* = 1.78) faces, *t*(921) = -2.30, *p* = 0.022, *d* = 0.08, whereas estimates for unaltered face height were greater for male (*M* = 0.02, *SD* = 1.49) than for female (*M* = -0.13, *SD* = 1.55) faces, *t*(921) = 2.09, *p* = 0.037, *d* = 0.07. However, both of these pairwise comparisons were nonsignificant after Bonferroni correction.

##### Jawbone prominence

There was a main effect for attribute level, *F*(2, 1842) = 44.30, *p* < 0.001, η^2^ = 0.05. Utility estimates were greater for masculinized (*M* = 0.35, *SD* = 1.15) than for unaltered (*M* = -0.31, *SD* = 1.32, *p* < 0.001, *d* = 0.30) and feminized (*M* = -0.04, *SD* = 1.23, *p* < 0.001, *d* = 0.20) jawbone prominence. Estimates were also higher for feminized than for unaltered jawbone prominence (*p* < 0.001, *d* = 0.12). There were no other significant main effects or interactions (all *F* < 1.14, *p* > 0.321).

## Discussion

The relative importance of five facial features (i.e., eyebrow thickness, cheekbone prominence, eye size, face height, and jawbone prominence) to perceptions of physical attractiveness and masculinity were assessed during participants’ evaluations of potential romantic partners’ facial photographs. CA was used to calculate individual facial feature importance values in overall rankings of attractiveness and masculinity and utility estimates for each attribute. Importance values for perceived masculinity were not significantly different for eyebrow thickness, face height, or jawbone prominence, but each of these traits was significantly greater than cheekbone prominence and eye size, suggesting that perceptions of physical masculinity are more strongly influences by eyebrow thickness, face height, and jawbone prominence compared to cheekbone prominence and eye size. Interestingly, this interacted with the sex of the face being ranked, whereby importance values for face height were greater for female faces compared to male faces. This indicates that a masculinized face height has a greater impact on the perceived masculinity of women’s faces than men’s faces. The opposite was true of jawbone prominence, which was perceptually more important in attributing masculinity to male faces than female faces. This latter finding should be interpreted with caution, however, as the effect fell short of significance after Bonferroni correction. Finally, utility estimates for masculinity rankings indicated that all features manipulated to appear more masculine or more feminine were ranked as such. Importantly, this indicates that the transformations were perceived as intended, further validating the use of computer graphics methods in objectively manipulating perceptions of sexual dimorphism (see also, e.g., Welling et al., 2007).

For physical attractiveness, these estimates were compared across (1) long- and short-term relationship contexts, and (2) sex of the face. With respect to physical attractiveness importance values, eyebrow thickness was not significantly more important than face height or jawbone prominence, but both eyebrow thickness and jawbone prominence were more important than cheekbone prominence and eye size when ranking faces for attractiveness. Likewise, importance values were greater for face height than for cheekbone prominence. In other words, participants in the current sample weighted the appearance of eyebrow thickness, face height, and jawbone prominence as most important in determining overall attractiveness, and eyebrow thickness and jawbone prominence as more important than eye size. Each of these traits appear to be important during zero-acquaintance assessments of physical attractiveness (Cunningham, 1986; Baudouin and Tiberghien, 2004), social dominance and maturity (Keating, 1985; Cunningham et al., 1990), and personality (Paunonen et al., 1999), however, this was the first study to examine which features are relatively more salient when digitally altered and presented alongside other digitally altered facial features within the same facial identity. Furthermore, no study has isolated the precise informational value of each particular facial feature to overall potential partner assessment. In this sense, it is difficult to conclude precisely why certain features were prioritized over others, although it opens up promising avenues for future investigation. Therefore, the informational value of each trait to perceptions of attractiveness may be best interpreted with respect to how preference for these traits varies across relationship context and sex of the face.

Importance values revealed that eye size was relatively more important for male versus female faces. Specifically, utility estimates showed that unaltered eye size was preferred more than masculinized or feminized eye size, and masculinized eye size was preferred more than feminized eye size, when collapsing across sex of face. However, masculinized (i.e., smaller) eye size was preferred more within short- versus long-term relationship contexts. One possibility is that eye size is an important attractiveness indicator in men insofar as individuals with smaller (i.e., masculinized) eyes are perceived as more mature or more socially dominant (Keating, 1985), which may make them more desirable as potential sexual (but not necessarily long-term) partners. Interestingly, unaltered eye size was preferred more in a long- compared to short-term context, perhaps suggesting that only a moderate level of masculinity/femininity is preferred in a long-term mate. However, these latter two findings fell short of significance after Bonferroni correction and should be interpreted with caution.

Importance values for face height were greater for female faces than for male faces, suggesting that the overall signal value of face height is more valuable for attributions of female attractiveness than for attributions of male attractiveness. Utility estimates revealed that masculinized face height was considered more attractive for male versus female faces, and, correspondingly, that feminized face height was preferred less for male versus female faces. Thus, sex-typical face height is preferred in both sexes, but the value of this trait in general is more salient in women’s faces compared to men’s, likely reflecting greater preferences for sex-typical women versus men (see Little et al., 2014). Similarly, utility estimates for masculinized jawbone prominence were greater for male than for female faces, and, correspondingly, estimates for feminized jawbone prominence were greater for female than for male faces, again suggesting that sex-typical traits are preferred more than sex-atypical traits. However, utility estimates were greater for masculinized compared to unaltered and feminized cheekbone prominence, regardless of the sex of the faces being ranked. Although sensible for male faces, this finding is unexpected for female faces, among whom one would expect a higher preference for feminized (i.e., more prominent) cheekbones. It is possible that participants simply preferred more masculine cheekbone prominence in both sexes, but this interpretation should be taken with caution because the highly subtle nature of this particular manipulation may have also made the differences more difficult to discern for the participants. That said, participants did accurately perceive masculinized cheekbone prominence as more masculine than unaltered or feminized cheekbone prominence, suggesting the manipulation was not imperceptibly subtle. This relationship should be investigated more thoroughly in future work.

Finally, although importance values for eyebrow thickness were greater for a long-term compared to short-term relationship context, this relationship was not significant after Bonferroni correction. Surprisingly, utility estimates revealed that thicker eyebrows were more attractive for female faces than male faces, particularly within a long-term mating context. Previous research shows that thicker eyebrows are typically perceived as more masculine and dominant (Windhager et al., 2011) and are more attractive in male than female faces (Keating, 1985), making this finding unexpected. It is possible that unmeasured personality features (e.g., sociosexuality, self-rated attractiveness) of the raters may moderate these findings. Indeed, recent work suggests that eyebrows may signal personality qualities (e.g., narcissism; Giacomin and Rule, 2018) that influence perceptions of attractiveness. Additionally, effect sizes in the current study were relatively small, and so investigation into the mediating effects of individual difference variables may further explain this relationship. Alternatively, it is possible that current and/or temporary cosmetic and style trends popular among the tested cohort are influencing this relationship, and so this relationship may not generalize to other populations. Certainly, this requires further investigation.

## Conclusion

This study is the first to demonstrate the utility of CA in investigating the importance of specific aspects or traits of a larger construct (e.g., facial masculinity) to the overall evaluation of that construct. Using this technique, we identified three traits (i.e., eyebrow thickness, jawbone prominence, and face height) whose digital manipulation appears to exert a relatively greater influence on perceptions of romantic partner attractiveness and masculinity than cheekbone prominence and eye size. We also showed how the relative salience of these traits shifts across the sex of the face and the relationship context (i.e., a long-term committed versus purely sexual relationship) for which they are evaluated. This contributes to a burgeoning literature of data-driven face analyses and promises to enrich the development of methodologies that allow researchers to study the relative contribution of distinct facial features to perceptually holistic facial representations.

## Ethics Statement

This study was carried out in accordance with the recommendations of the Oakland University Institutional Review Board with written informed consent from all subjects. All subjects gave written informed consent in accordance with the Declaration of Helsinki. The protocol was approved by the Oakland University Institutional Review Board.

## Author Contributions

JM was responsible for devising the study design, data collection and analysis, and manuscript preparation. LW provided advisory support and guidance throughout each step of this project.

## Conflict of Interest Statement

The authors declare that the research was conducted in the absence of any commercial or financial relationships that could be construed as a potential conflict of interest.
